# A bacterial binary toxin system that kills both insects and aquatic crustaceans: *Photorhabdus* insect-related toxins A and B

**DOI:** 10.1371/journal.ppat.1011330

**Published:** 2023-05-04

**Authors:** Hao-Ching Wang, Shin-Jen Lin, Han-Ching Wang, Ramya Kumar, Phuoc Thien Le, Jiann-Horng Leu

**Affiliations:** 1 Graduate Institute of Translational Medicine, College of Medical Science and Technology, Taipei Medical University, Taipei, Taiwan, Republic of China; 2 The PhD Program for Translational Medicine, College of Medical Science and Technology, Taipei Medical University and Academia Sinica, Taipei, Taiwan, Republic of China; 3 International Center for the Scientific Development of Shrimp Aquaculture, National Cheng Kung University, Tainan, Taiwan, Republic of China; 4 Department of Biotechnology and Bioindustry Sciences, College of Bioscience and Biotechnology, National Cheng Kung University, Tainan, Taiwan, Republic of China; 5 Institute of Marine Biology, National Taiwan Ocean University, Keelung, Taiwan, Republic of China; 6 Center of Excellence for the Oceans, National Taiwan Ocean University, Keelung, Taiwan, Republic of China; University of Basel, SWITZERLAND

## Abstract

*Photorhabdus* insect-related toxins A and B (PirA and PirB) were first recognized as insecticidal toxins from *Photorhabdus luminescens*. However, subsequent studies showed that their homologs from *Vibrio parahaemolyticus* also play critical roles in the pathogenesis of acute hepatopancreatic necrosis disease (AHPND) in shrimps. Based on the structural features of the PirA/PirB toxins, it was suggested that they might function in the same way as a *Bacillus thuringiensis* Cry pore-forming toxin. However, unlike Cry toxins, studies on the PirA/PirB toxins are still scarce, and their cytotoxic mechanism remains to be clarified. In this review, based on our studies of *V*. *parahaemolyticus* PirA^*vp*^/PirB^*vp*^, we summarize the current understanding of the gene locations, expression control, activation, and cytotoxic mechanism of this type of toxin. Given the important role these toxins play in aquatic disease and their potential use in pest control applications, we also suggest further topics for research. We hope the information presented here will be helpful for future PirA/PirB studies.

## 1. Introduction

*Photorhabdus luminescens* is an entomopathogenic bacterium that has the ability to kill a wide range of insects **[1,2]**. This bacterium kills the insects by secreting various toxins, such as the toxin complexes (Tcs) and the “makes caterpillars floppy” (Mcf) toxins **[1,2]**. *Photorhabdus* insect-related toxins A and B (PirA and PirB) are one of the insecticidal toxins secreted by *P*. *luminescens* **[2]**. In addition to *P*. *luminescens*, the relevant genes have also been found in many other bacteria, including *Xenorhabdus* spps. and *Vibrio* spps. Notably, the *Vibrio parahaemolyticus* PirA/PirB homologs (referred to here as PirA^*vp*^ and PirB^*vp*^) were found to be the key pathogenetic factors of acute hepatopancreatic necrosis disease (AHPND) in shrimps **[3]**. We note that both insecticidal *P*. *luminescens* and marine pathogenic *V*. *parahaemolyticus* have their own *pirA/pirB* genes and that this suggests the functional importance of this toxin family during evolution. However, despite their evident importance, there are still relatively few studies on the PirA/PirB toxins, especially on their cytotoxic mechanisms. In this review, we summarize our current understanding of this toxin family in light of the findings provided by recent reports. We also identify topics that we feel deserve particular attention and hope that this will be helpful for future studies.

## 2. Insecticidal PirA/PirB

*Photorhabdus* PirA/PirB toxins were first determined from the *P*. *luminescens* W14 genome **[1]**. In *Photorhabdus*, *Sodalis*, *Xenorhabdus*, and *Yersinia* spps., the 2 toxins are encoded by 2 distinct, but adjacent loci on the bacterial genome (Table 1). These *pirA*/*pirB* genes are both on the same operon, suggesting that they are co-regulated and co-transcribed. Furthermore, a comparison of the genomic organization of *pirA/pirB* operons indicated that some phage genes are found in the *Yersinia* Pir locus and that there is a Fis-type DNA recombinase adjacent to the *Photorhabdus* operons **[15]**. This suggests that these *pirA/pirB* genes have been acquired by horizontal transmission **[15]**.

**Table 1:** The gene locations of PirA/PirB toxins.

Species	*pirA*	*pirB*	Location
*Photorhabdus asymbiotica*	WP015835800.1 (aa)	WP015835799.1 (aa)	Genomic DNA [4]
*Photorhabdus luminescens*	ABE68878.1 (aa)	ABE68879.1 (aa)	Genomic DNA [5]
*Sodalis praecaptivus*	AHF77486.1 (aa)	AHF77485.1 (aa)	Genomic DNA (NCBI database)
*Xenorhabdus cabanillasii*	CDL79383.1 (aa)	CDL79384.1 (aa)	Genomic DNA (NCBI database)
*Xenorhabdus doucetiae*	CDG18638.1 (aa)	CDG18639.1 (aa)	Genomic DNA (NCBI database)
*Xenorhabdus nematophila*	WP013183676.1 (aa)	WP010845483.1 (aa)	Genomic DNA [6]
*Yersinia enterocolitica*	ANI30071.1 (aa)	ANI30072.1 (aa)	Genomic DNA [7]
*Algoriphagus sp*.	QNH91014.1 (aa)	QNH91015.1 (aa)	Plasmid. *Algoriphagus* sp. can acquire the plasmid containing *pirAB* genes from *V*. *parahaemolyticus* via conjugation [8]
*Vibrio campbellii*	QOE77699.1 (aa)	QOE77700.1 (aa)	Plasmid [9,10]
*Vibrio harveyi*	QOE77701.1 (aa)	QOE77702.1 (aa)	Plasmid [9,11]
*Vibrio owensii*	AQT24297.1 (aa)	AQT24298.1 (aa)	Plasmid [9,12]
*Vibrio parahaemolyticus*	WP023622799.1 (aa)	WP025789543.1 (aa)	Plasmid [3]
*Vibrio punensis*	NCBI Genome Database under the accession number PRJNA412371		Plasmid [13]
*Alcaligenes faecalis*	WP003801867.1 (aa)	WP003801865.1 (aa)	Unknown (NCBI database)
*Micrococcus luteus*	MH046196.1 (nt)	MH046196.1 (nt)	Unknown [14]
*Vibrio alginolyticus*	QOE77705.1 (aa)	QOE77706.1 (aa)	Unknown (NCBI database)
*Vibrio sinaloensis*	QOE77707.1 (aa)	QOE77708.1 (aa)	Unknown (NCBI database)

It was initially proposed that *Photorhabdus* PirA/PirB was an insect JHE (juvenile hormone esterase)-like protein that disrupted insect metamorphosis by catalyzing the hydrolysis of juvenile hormone (JH) **[5]**. However, a subsequent study found that *Photorhabdus* PirA/PirB did not have JHE enzymatic activity **[4]**. Amino acid sequence analysis then suggested that *Photorhabdus* PirB, but not PirA, had some similarity to the pore-forming domain of *Bacillus thuringiensis* Cry toxin **[16]**. The resemblance between PirA/PirB and Cry toxin was further confirmed by a structural analysis of the PirA/PirB homologs (PirA^*vp*^ and PirB^*vp*^) from the marine pathogen *V*. *parahaemolyticus* **[3,17]**.

PirA and PirB together form a binary toxin system. This kind of system usually consists of 2 components with different functionality; typically, one component has cytotoxic activity while the other mediates binding to a specific receptor **[18]**. A number of studies have demonstrated the lethality of the PirA/PirB system. For example, histological examination of *Plutella xylostella* larvae fed with *Escherichia coli* expressing recombinant PirA/PirB toxins from *P*. *luminescens* showed a profound swelling and shedding of the apical membranes of the midgut epithelium **[19]**. A fusion PirAB toxin from *P*. *asymbiotica* was also shown to be toxic to the larvae of *Aedes aegypti*, *Aedes albopictus* **[15]**, and *Spodoptera exigua* **[20]**. Full toxicity is also thought to require the presence of both PirA and PirB **[3]**. Thus, in an assay of *X*. *nematophila* PirA/PirB toxicity to the caterpillar of wax moth *Galleria mellonella*, the injection of either PirA or PirB alone only resulted in low mortality rates compared to the injection of both PirA and PirB in combination **[6]**. All of these results support the conclusion that PirA/PirB toxins complement each other to maximize their insecticidal activity.

## 3. AHPND-causing PirA^*vp*^/PirB^*vp*^

AHPND, or shrimp early mortality syndrome (EMS) as it has previously been called, induces major hepatopancreatic cell damage in the diseased shrimp **[3,17,21–23]**. The damaged hepatopancreatic tissue has a pale-to-white color and loses its digestive and immune functions **[3,17,21–23]**. In farmed shrimps such as *Litopenaeus vannamei* and *Penaeus monodon*, AHPND can cause epidemics with high mortality (70% to 100%) **[24]**. After AHPND/EMS first emerged in China in 2009 **[25]**, it rapidly spread across Asian countries such as Thailand, the Philippines, Vietnam, Malaysia, and Bangladesh **[26]**, and since 2019, it has also been making an impact in the United States and Mexico **[25,27]**. Due to the sharp decline in global farmed shrimp production caused by AHPND **[24]**, this disease has come to the attention of the United Nations and the World Organization for Animal Health.

In 2013, although there were no previous reports suggesting that *V*. *parahaemolyticus* could cause such severe epidemics in shrimps, specific strains of this halophilic, gram-negative bacterium were identified as the pathogenic source of AHPND **[21]**. Until that time, *V*. *parahaemolyticus* was known primarily for being an opportunistic pathogen that causes human gastroenteritis and other symptoms after the consumption of insufficiently fresh seafood **[28]**. In 2014, Yang and colleagues used a high-throughput next-generation sequencing (NGS) platform to compare the differences in AHPND pathogenic and nonpathogenic *V*. *parahaemolyticus* genome sequences **[29]**. Surprisingly, the pathogenic genes were not found in the chromosome, and it was shown instead that all the pathogenic *V*. *parahaemolyticus* strains contained a plasmid, pVA1, with an approximate size of 70 kb **[3,22,29]**. Gene annotation of pVA1 showed that it contained about 40 functional genes, including transposase, binding factor, anti-restriction protein, type II and type III secretion systems, and 2 *Photorhabdus* insect-related (Pir) binary toxins (PirA^*vp*^ and PirB^*vp*^) **[22]**. In other studies, PirA^*vp*^ and PirB^*vp*^ were confirmed as the key factors for AHPND symptoms, i.e., the hepatopancreatic cell damage **[3]**. Because of their functional importance in AHPND pathogenesis, PirA^*vp*^/PirB^*vp*^ have been considered potential targets for drug therapy **[17,30]**.

In addition to *V*. *parahaemolyticus*, *pirA*^*vp*^/*pirB*^*vp*^ like-genes have also been found in other *Vibrio* spps. **[9–13]**. However, unlike *Photorhabdus* and *Xenorhabdus*, *Vibrio pirA/pirB* genes were usually found in the plasmid, not in the genomic DNA **[9–13]**. As shown in Table 1, 5 different *Vibrio* species have so far been confirmed to contain *pirA/pirB* genes in their plasmids. Several studies have revealed that different *Vibrio* species may transfer pVA1-like plasmid from an AHPND-causative strain to a non-AHPND-causative strain through horizontal transfer **[31–34]**. It should also be noted that transposase genes are usually found around the *Vibrio pirA/pirB* genes **[3,9,22,35–36]**. For example, in the pVA1 plasmid isolated from *V*. *parahaemolyticus* strain 3HP, the *pirA/pirB-*containing gene cluster (ORF 49–54) is flanked by 2 transposase genes in opposite directions (ORF 48 and ORF 55) **[3]**. The inverted repeats in the terminals of these 2 transposase genes suggest that the *pirA*^*vp*^/*pirB*^*vp*^ gene cluster came from a transposon-mediated horizontal transfer. Similarly, 2 transposase genes were found at both ends of the *pirA*^*vp*^/*pirB*^*vp*^ gene cluster in the pVH plasmid from AHPND-causing *V*. *owensii* **[9]**. Transposon genes can also interfere with the expression of *pirA*^*vp*^/*pirB*^*vp*^. Thus in the pVA1-like plasmid isolated from *V*. *parahaemolyticus* strain XN87, an out-of-frame insertion caused by a transposon gene fragment was found to prevent the expression of the downstream *pirA*^*vp*^/*pirB*^*vp*^ gene **[35]**. The *pirA*^*vp*^/*pirB*^*vp*^ expression was also prevented in another pVA1-like plasmid from a non-AHPND *V*. *parahaemolyticus* strain (R14), which has an inserted transposon gene in the *pirA*^*vp*^/*pirB*^*vp*^ promoter region **[36]**. All of these examples demonstrate the importance of transposase in the transfer, deletion, and mutation of the *pirA*^*vp*^/*pirB*^*vp*^*-*containing gene cluster in *Vibrio* spps.

## 4. Structural similarities indicate that PirA/PirB toxins are Cry-like pore-forming toxins

**Fig 1:**
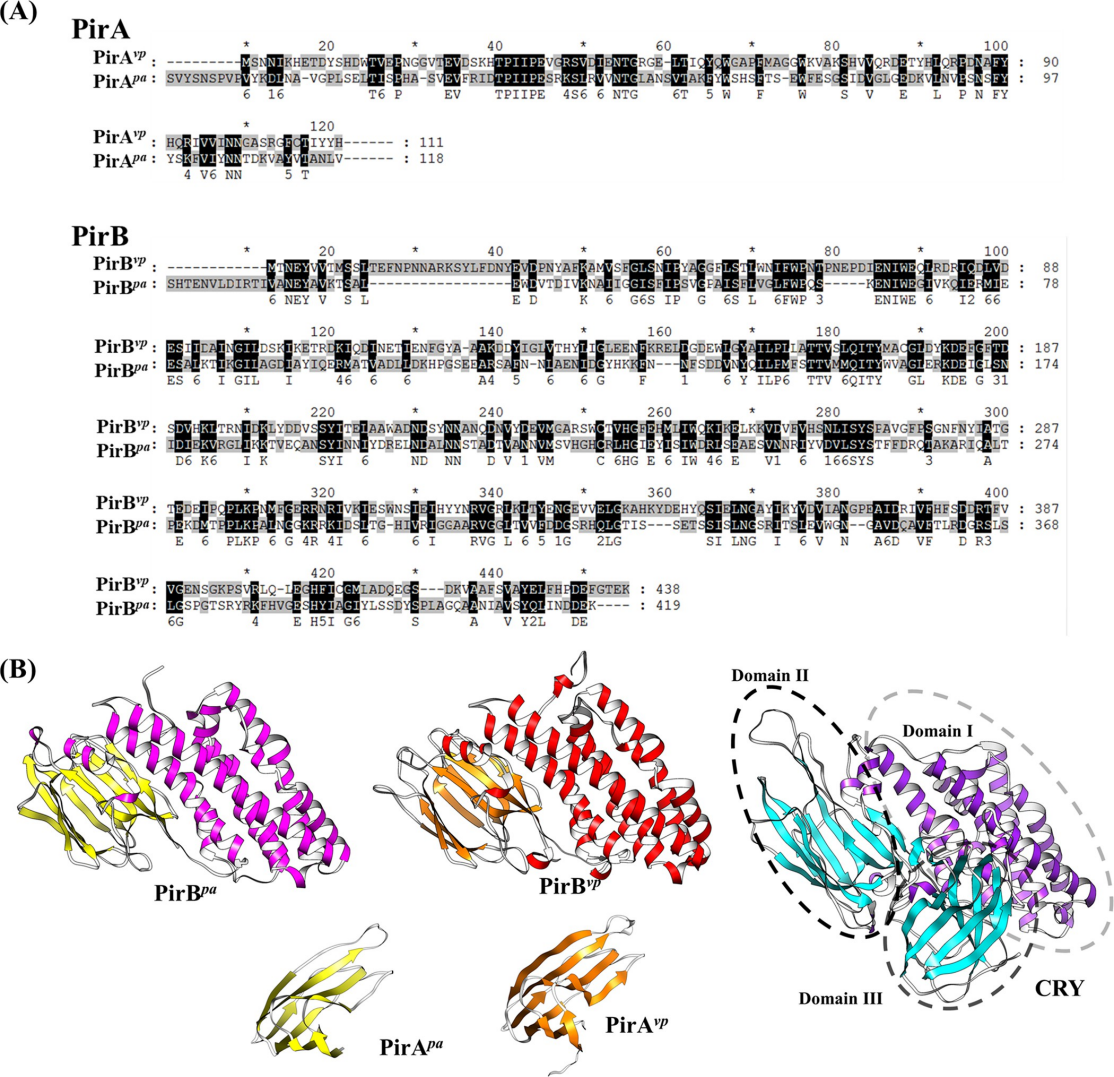
A comparison of the amino acid sequences (A) and structures of PirA^*vp*^/PirB^*vp*^ and PirA^*pa*^/PirB^*pa*^ (B). The sequence alignment was performed using Clustal Omega (https://www.ebi.ac.uk/Tools/msa/clustalo/), and UCSF Cherima (https://www.cgl.ucsf.edu/chimera/) was used to present the toxin structures. The PDB codes 3X0T, 7FCN, 3X0U, 7FCDP, and 1CIY were used to produce the figures for PirA^*vp*^, PirA^*pa*^, PirB^*vp*^, PirB^*pa*^, and Cry toxins, respectively.

*V*. *parahaemolyticus* PirA^*vp*^/PirB^*vp*^ were the first structures to be determined from this toxin family **[3]**. In 2022, the structures of insecticidal PirA/PirB (*Photorhabdus akhursti* PirA*/*PirB; PirA^*pa*^/PirB^*pa*^) have also been released to the protein data bank (PDB DOI: 10.2210/pdb7FCN/pdb and 10.2210/pdb7FDP/pdb). Although there are some differences in the amino acid sequences, a comparison of the structures of PirA^*vp*^ to PirA^*pa*^ and PirB^*vp*^ to PirB^*pa*^ show that they are highly similar (Fig 1A and 1B). We interpret this to mean that during their evolution, these toxins remained functionally conserved.

*Bacillus* Cry-like toxins belong to a family of insecticidal toxins that kill insects by forming pores on the membrane of midgut cells **[37–39]**, so the structural similarity to *Bacillus thuringiensis* Cry toxins further suggests that PirA/PirB toxins are also pore-forming toxins **[3,17,22]**. Structural analysis reveals that the cytotoxic mechanism of the Cry toxins is achieved by 3 functional domains. From the N-terminal to the C-terminal, Cry domain I has pore-forming activity, domain II is used for receptor binding, and domain III is for sugar recognition **[37–39]**. These domains implement the cytotoxic mechanism of Cry as follows: when Cry toxins reach the midguts of insect larvae, Cry domain III binds to the sugars (e.g., N-Acetylgalactosamine, GalNAc) on the target receptors (e.g., aminopeptidase N, APN; alkaline phosphatase, ALP; cadherin-like receptor, CAD). Domain II then further binds to the target receptors, until finally, the protease activity of the toxin-bound receptors mediates a proteolytic cleavage of the α1 helix of Cry domain I. This cleavage triggers a conformational change of Cry toxin to an active pore-forming state. A subsequent oligomeric form of Cry toxin results in uncontrolled pores on the cell membrane, which in turn leads to cell death by osmotic lysis **[37–39]**.

Structural comparisons show that PirA toxins have structural features that are similar to Cry domain III, while the N-terminal and C-terminal of PirB toxins are respectively similar to Cry domains I and II (Fig 1B) **[3,17,40]**. A reasonable sequence for the cytotoxic mechanism of PirA/PirB would therefore be: first, the formation of a complex that contains the full set of functional domains; the next step would be binding to the respective sugar/receptor on the cell surface; and finally, this would trigger a conformational change that activates the transmembrane activity of PirB, leading to the formation of a higher order oligomer that creates uncontrolled pores.

## 5. The expression control, activation, and cytotoxic mechanism of PirA/PirB toxins

While the mechanisms described above provide a plausible outline of how the PirA/PirB toxins might effectively act as *Bacillus* Cry-like, pore-forming toxins, a more detailed understanding of the toxins’ expression control, activation, and cytotoxic mechanisms still needs to be elucidated. Among the various PirA/PirB toxins, the cellular mechanisms of AHPND-causing PirA^*vp*^/PirB^*vp*^ are relatively well studied, so in this section, we summarize these reports and propose various respective mechanisms.

### 5.1. The activation and expression control of PirA^*vp*^/PirB^*vp*^ toxins

Outbreaks of AHPND are known to be influenced by environmental stressors like temperature, pH, and water quality **[41–43]**. There are also reports that the expression of the *pirA*^*vp*^/*pirB*^*vp*^ genes is directly affected by environmental changes. For example, *pirA*^*vp*^/*pirB*^*vp*^ gene expression was increased when the temperature shifted from a higher to a lower range (26°–32°C to 22°–28°C) **[44]**. Apart from water temperature, salinity also plays a key role in regulating the metabolism and virulence gene expression in *V*. *parahaemolyticus* **[45,46]**, with the gene expression levels of *pirA*^*vp*^ found to be up-regulated when salinity increased significantly. Since its expression is affected by salinity, it has been proposed that transcription of the *pirA*^*vp*^ gene might be Na^+^ dependent **[47]**. The presence of bile acids, which are commonly used as a feed additive for shrimps, was also reported to induce PirA^*vp*^/PirB^*vp*^ toxin release and biofilm formation in AHPND-causing *V*. *parahaemolyticus* **[23,48]**.

Another recent report further suggested that the mRNA and protein expressions of *pirA*^*vp*^/*pirB*^*vp*^ were related to the quorum sensing (QS) system and low pH **[49]**. QS is a cell density-dependent process that is seen in many bacteria, including *V*. *harveyi*, *V*. *cholerae*, and *V*. *parahaemolyticus* **[50]**. The system is involved in many physiological processes that respond to environmental stress, such as biofilm formation, bioluminescence, conjugation, plasmid transfer, antibiotic production, cell mobility, and sporulation **[51]**. Notably, the QS system also regulates the expression of virulence factors, such as the type III secretion systems, type VI secretion systems, and the thermostable direct hemolysin genes *tdh1* and *tdh2* **[52–54]**. Lin and colleagues showed that an important regulator of the QS system, LuxO^*vp*^, is a negative regulator of the *pirA*^*vp*^ and *pirB*^*vp*^ toxin genes **[49]**. In addition, a transcription factor in the QS system, AphB^*vp*^, was confirmed to bind with the predicted promoter region of *pirA*^*vp*^/*pirB*^*vp*^ **[49]**. In *V*. *cholerae* and *V*. *alginolyticus*, AphB has been shown to be a key virulence regulator **[55,56]**, and in *V*. *cholerae*, downstream genes regulated by AphB are also important for bacterial survival in low pH conditions and anaerobic environments **[57]**. If AphB is able to respond to environmental changes by altering its DNA-binding ability to regulate these downstream genes, it follows that the regulation of *pirA*^*vp*^/*pirB*^*vp*^ genes by AphB^*vp*^ may also be pH dependent.

It seems clear that environmental factors, especially environmental pH, play important roles in the control of *pirA*^*vp*^/*pirB*^*vp*^ gene expression. It would be interesting to investigate the extent to which the gene/protein expression of insecticidal PirA/PirB might similarly be affected by environmental factors, but this has not yet been explored.

### 5.2. The cytotoxic mechanism of PirA^*vp*^ and PirB^*vp*^ toxins

Further to the suggestion in Section 4 that PirA/PirB toxins might act as Cry toxins, we now further consider details of the Cry-like cytotoxic mechanisms that might be used by the PirA^*vp*^ and PirB^*vp*^ toxins. We have to state here, however, that although we used Cry toxin as a model to propose a mechanism for PirA^*vp*^/PirB^*vp*^, we cannot be sure that the mechanism of PirA^*vp*^/PirB^*vp*^ is exactly the same as Cry toxin because the research is still in progress. Nevertheless, based on the available information, we believe that the most suitable reference model for PirA^*vp*^/PirB^*vp*^ so far is Cry toxin, and that it provides a useful guide to future experiments and thinking.

#### 5.2.1. Forming a Cry-like three-domain toxin

To begin with, the 2 toxins need to be capable of assembly into a Cry-like, three-domain conformation. In the case of the PirA^*vp*^/PirB^*vp*^ toxins, this kind of physiological binding is supported by several reports **[3,40]**. Specifically, in 2019, Lin and colleagues determined that PirA^*vp*^ and PirB^*vp*^ formed a hetero-tetramer in a 1:1 binding stoichiometry by using gel filtration and densitometric analysis and further proposed a PirA^*vp*^/PirB^*vp*^ hetero-tetramer model by using hydrogen/deuterium exchange mass spectrometry **[40].** In this model, the PirA^*vp*^/PirB^*vp*^ complex contains 2 copies of the Cry-like three-domain toxin that supports the pore-forming activity. Interestingly, the binding affinity between PirA^*vp*^ and PirB^*vp*^ in solution is not strong, but only moderate to weak (approximately 7 μm, as measured by isothermal titration calorimetry) **[40]**. This would lead to an instability in the PirA^*vp*^/PirB^*vp*^ complex, but since the above experiments were carried out in the absence of membrane receptors or sugars, it is possible that an interaction with the receptor/sugar itself may be necessary to improve the stability of the PirA^*vp*^/PirB^*vp*^ complex. Another possibility is that this binding instability of the PirA^*vp*^/PirB^*vp*^ complex may be a control mechanism that reduces their combined cytotoxicity by complex dissociation. Although several details still need to be explored, it remains clear that PirA^*vp*^ and PirB^*vp*^ do indeed have the ability to form a Cry-like three-domain toxin before binding to their receptors.

#### 5.2.2. Binding to sugars/receptors on the cell membrane

After forming a Cry-like toxin, PirA/PirB would be expected to target its respective sugars/receptors to attach to the cell membrane. In 2021, Luangtrakul and colleagues reported that shrimp aminopeptidase N receptors are responsible for binding PirA^*vp*^ and PirB^*vp*^. In *hemocytes* of *L*. *vannamei*, 2 aminopeptidase N (APN) proteins were identified, *Lv*APN1 and *Lv*APN2, with the membrane-bound *Lv*APN1 being shown to act as a receptor for the PirAB^*vp*^ toxins **[58]**. APN, which is a membrane protein found in many species, is an enzyme with broad substrate specificity that can remove the N-terminal amino acid from oligopeptides and proteins **[59]**, and it has previously been noted that the binding between Cry toxin and APN in an insect’s midgut plays an important role in Cry toxin activation **[38,39]**. Luangtrakul and colleagues found not only that the expression of *Lv*APN1 was increased significantly after treatment with AHPND-causing *V*. *parahaemolyticus* or PirA^*vp*^/PirB^*vp*^ toxins, but also that truncated *Lv*APN1 recombinant protein interacted with recombinant PirA^*vp*^ and PirB^*vp*^ toxins in a dose-dependent manner **[58]**. Furthermore, in *Lv*APN1 knockdown shrimps, toxin-induced mortality, clinical symptoms in the hepatopancreas, and the number of AHPND-causing *V*. *parahaemolyticus* bacteria were all reduced **[58]**. Interestingly, when PirAB^*vp*^ toxins were used to challenge the *Lv*APN1-knockdown shrimp, PirB^*vp*^ toxin was found to be distributed only on the membrane of hemocytes and could not be internalized into the cytoplasm. Another report showed that recombinant PirB^*vp*^ toxin could interact with histones, resulting in the dephosphorylation of Serine 10 in histone H3, and the subsequent induction of apoptosis **[60]**. Together, these results suggest that PirB^*vp*^ toxins may translocate into the cell through direct interaction with *Lv*APN1, after which they further enter the nucleus and cause hemocyte damage.

In addition to *Lv*APN1, alpha amylase-like protein was also reported as a potential receptor for PirB^*vp*^ **[61]**. Functionally, alpha amylase-like protein has 1,4-α-D-glucan glucanohydrolase activity, and it was identified as a receptor for *B*. *thuringiensis* ssp Cry4Ba and Cry11Aa toxins **[62]**. Two other glycoproteins, beta-hexosaminidase subunit beta and mucin-like 5 AC, which are expressed in the epithelial cells of the shrimp’s hepatopancreas, were also found to interact with PirB^*vp*^ **[63]**. These studies all strengthen the hypothesis that PirB^*vp*^ has receptor-binding ability. In the future, site mutation could be used to further identify which regions of PirB^*vp*^ (e.g., the loop regions of C-terminal domain of PirB^*vp*^) might mediate receptor binding. Meanwhile, we also note that there are slight differences in the receptor binding region of the C-terminal of PirB^*vp*^ and PirB^*pa*^ (Fig 1B). This might mean that the target receptors of PirB^*pa*^ may not be identical to those of PirB^*vp*^, but it is more likely to reflect the fact that the same PirA/PirB receptors in insects and shrimps have evolved into slightly different structures, so that different interfaces are required for efficient binding to these homologous receptors.

Different from the Cry toxins, PirB^*vp*^ has been found to interact with a fatty acid binding protein (FABP) of *L*. *vannamei* directly. Knocking down of *Lv*FABP reduced shrimp mortality and histopathological signs of AHPND, as well as the number of the AHPND-causing *V*. *parahaemolyticus* in the challenged shrimp **[64]**. FABPs may occur as membrane associated or cytosolic proteins, whereas the cell localization of *Lv*FABP is still unclear. Therefore, it remains to be further investigated whether *Lv*FABP can be a receptor of PirA^*vp*^/PirB^*vp*^ toxin **[64]**.

Compared to its ability to bind to receptors, the ability of PirA/PirB to bind to sugars is not so clear. To date, even though structural similarities to Cry domain III suggest that PirA^*vp*^ may function as a sugar-binding protein **[3,17]**, this has still not been confirmed experimentally. Interestingly, a recent report indicated that PirB^*vp*^, but not PirA^vp^, is able to target glycosaminoglycans like GalNH_2_ and GlcNH_2_ **[65]**, suggesting that PirB^*vp*^ is able to bind both receptors and sugars. This putative sugar-binding ability of PirB^*vp*^ has also been indirectly confirmed by the binding between PirB^*vp*^ and glycoproteins **[63]**. We note, however, that the sugar-binding activity evidently does not play a critical role for the interaction between *Lv*APN1 and PirA^*vp*^/PirB^*vp*^, since the *Lv*APN1 used for the ELISA assay was prepared from *E*. *coli* and was not glycosylated **[58]**. Taken together, the functional roles of PirA^vp^, as well as the sugar-binding abilities of PirA^*vp*^/PirB^vp^, remain to be further investigated.

#### 5.2.3. Forming transmembrane pores by oligomerization

The final stage of the cytotoxic mechanism of a pore-forming toxin is to create unregulated pores in the cell membrane. After attaching to the cell membrane by binding to a receptor, pore-forming toxins undergo a conformational change to expose their hydrophobic regions. The resulting toxin is no longer water soluble, but instead becomes an oligomeric transmembrane pore that inserts into the cell membrane **[66]**. These conformational changes and the oligomerization of pore-forming toxins have been widely studied **[66–69]**. For example, structural studies of Cytolysin A (ClyA), which is a pore-forming toxin found in several enterobacteria, have revealed both water-soluble monomeric and pore-forming dodecameric conformations **[70–72]**.

Unfortunately, however, transmembrane pore conformation is not easily investigated, and we do not yet have a good understanding of the transmembrane pore conformation and oligomerization of PirA^*vp*^/PirB^*vp*^. Over the past decade, although structural approaches including X-ray crystallography and CryoEM have been used to reveal the transmembrane pore conformations of toxins such as ClyA and Aerolysin **[72,73]**, the currently reported structures for PirA^*vp*^/PirB^vp^ were all derived from their water-soluble forms, not their transmembrane forms **[3,40]**. Furthermore, although PirA^*vp*^/PirB^*vp*^ toxins are able to form a hetero-tetramer in solution **[40]**, this does not reflect the real conformation of the toxins after binding to the membrane.

To investigate the transmembrane stage of novel pore-forming toxins like PirA^*vp*^/PirB^*vp*^, 2 main challenges need to be overcome. The first challenge is to determine the activation conditions. In the case of PirA^*vp*^/PirB^*vp*^’s reference, i.e., the Cry toxins, activation is triggered by the proteolytic cleavage of the N-terminal peptide of domain I **[38,39]**, which causes the activated Cry toxin to reconfigure into a transmembrane state. However, the process that initiates this mechanism for PirA^*vp*^/PirB^*vp*^ remains to be elucidated. The second challenge is to create a suitable membrane-like platform for PirA^*vp*^/PirB^*vp*^ to bind and insert into. For this purpose, detergents have often been used to mimic a membranous environment. For example, 0.1% n-Dodecyl-beta-Maltoside (DDM) was used to induce the formation of 13-mers of the *E*. *coli* Cytotoxin ClyA **[70]**. Determining the state of PirA^*vp*^/PirB^*vp*^ in different detergents will be an essential first step in this research.

**Fig 2:**
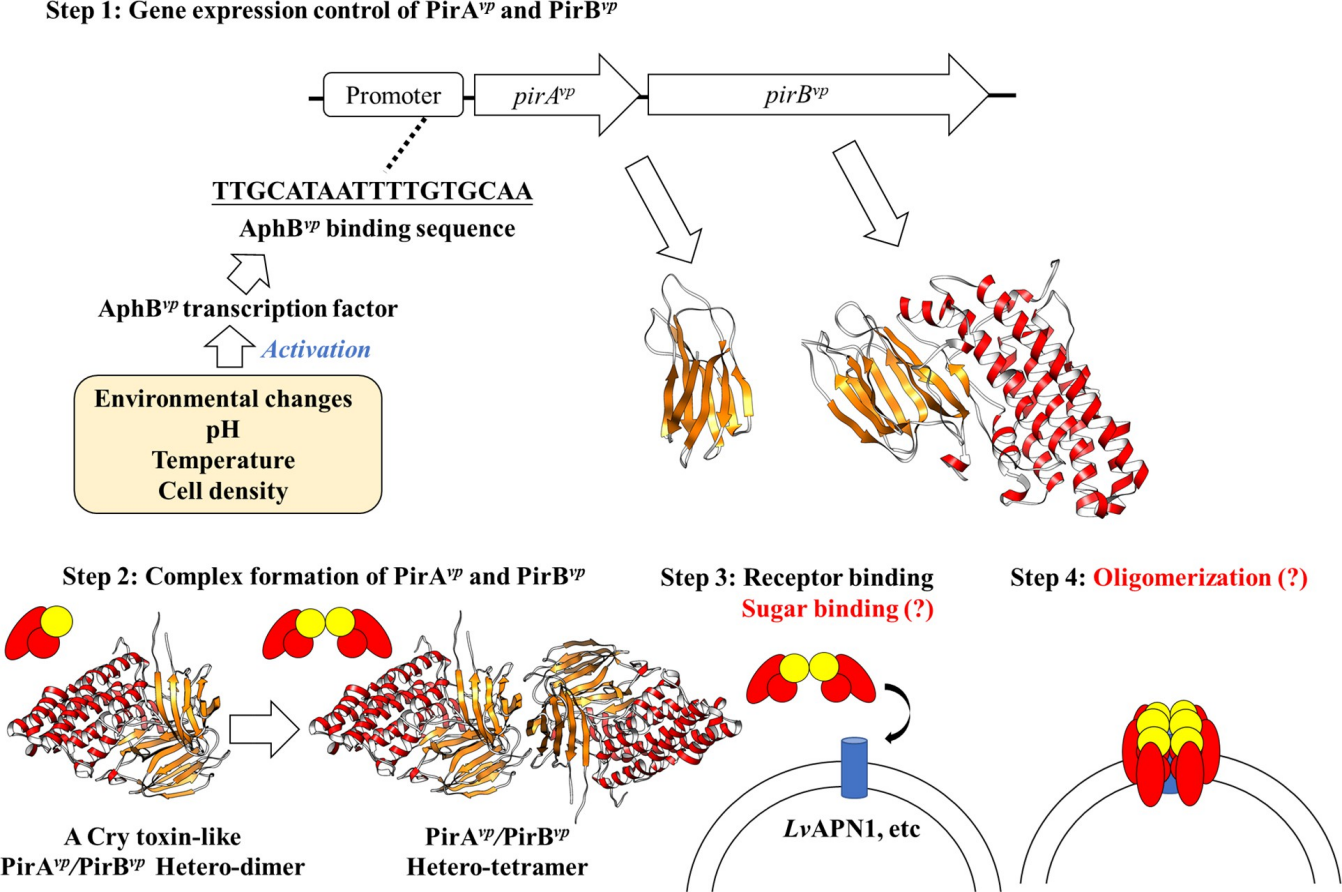
Current understanding of the cytotoxic mechanisms of the PirA^*vp*^ and PirB^*vp*^ toxins.

Our current understanding of these mechanisms therefore suggests a possible cytotoxic mechanism for the PirA/PirB toxins, and particularly for PirA^*vp*^/PirB^*vp*^, as summarized in Fig 2. Details of the sugar-binding and pore-forming mechanisms will need to be further investigated, but we also note that future PirA^*vp*^/PirB^*vp*^ studies could in turn provide a useful research reference for insecticidal PirA/PirB.

## Concluding remarks

As this review has shown, our current understanding of the PirA/PirB toxins is derived from only a limited number of studies, and many mechanisms remain to be elucidated. Meanwhile, these toxins are already deployed in a range of agricultural applications, and further research on this toxin family will be important not just for these insecticidal toxins (e.g., *P*. *luminescens* PirA/PirB), but also for potential applications in the prevention and treatment of the shrimp disease AHPND. We hope that this review will help to encourage future study and increase our knowledge of this under-researched toxin family.
